# Proinsulin peptide promotes autoimmune diabetes in a novel HLA-DR3-DQ2-transgenic murine model of spontaneous disease

**DOI:** 10.1007/s00125-019-04994-8

**Published:** 2019-10-14

**Authors:** Johan Verhagen, Norkhairin Yusuf, Emma L. Smith, Emily M. Whettlock, Kerina Naran, Sefina Arif, Mark Peakman

**Affiliations:** 1 School of Immunology and Microbial Sciences, Faculty of Life Sciences & Medicine, King’s College London, 2nd Floor Borough Wing, Guy’s Hospital, Great Maze Pond, London, SE1 9RT UK; 2 UCB Pharma Ltd, Slough, UK; 3 Present Address: Department of Metabolism, Digestion and Reproduction, Chelsea & Westminster Hospital, London, UK; 4 Institute of Diabetes, Endocrinology and Obesity, King’s Health Partners, London, UK

**Keywords:** HLA-DQ2, HLA-DR3, Mouse model, Proinsulin, Type 1 diabetes

## Abstract

**Aims/hypothesis:**

The molecular basis for the pathological impact of specific HLA molecules on autoimmune diseases such as type 1 diabetes remains unclear. Recent natural history studies in children have indicated a link between specific HLA genotypes and the first antigenic target against which immune responses develop. We set out to examine this link in vivo by exploring the diabetogenicity of islet antigens on the background of a common diabetes-associated HLA haplotype.

**Methods:**

We generated a novel HLA-transgenic mouse model that expresses high-risk genes for type 1 diabetes (DRB1*03:01-DQA1*05:01-DQB1*02:01) as well as human *CD80* under the rat insulin promoter and human *CD4*, on a C57BL/6 background. Adjuvanted antigen priming was used to reveal the diabetogenicity of candidate antigens and peptides.

**Results:**

HLA-DR3-DQ2^+^huCD4^+^IA/IE^−/−^RIP.B7.1^+^ mice spontaneously developed autoimmune diabetes (incidence 46% by 35 weeks of age), accompanied by numerous hallmarks of human type 1 diabetes (autoantibodies against GAD65 and proinsulin; pancreatic islet infiltration by CD4^+^, CD8^+^ B220^+^, CD11b^+^ and CD11c^+^ immune cells). Disease was markedly accelerated and had deeper penetrance after adjuvanted antigen priming with proinsulin (mean onset 11 weeks and incidence 100% by 20 weeks post challenge). Moreover, the diabetogenic effect of proinsulin located to the 15-residue B29-C11 region.

**Conclusions/interpretation:**

Our study identifies a proinsulin-derived peptide region that is highly diabetogenic on the HLA-DR3-DQ2 background using an in vivo model. This approach and the peptide region identified may have wider implications for future studies of human type 1 diabetes.

**Electronic supplementary material:**

The online version of this article (10.1007/s00125-019-04994-8) contains peer-reviewed but unedited supplementary material, which is available to authorised users.

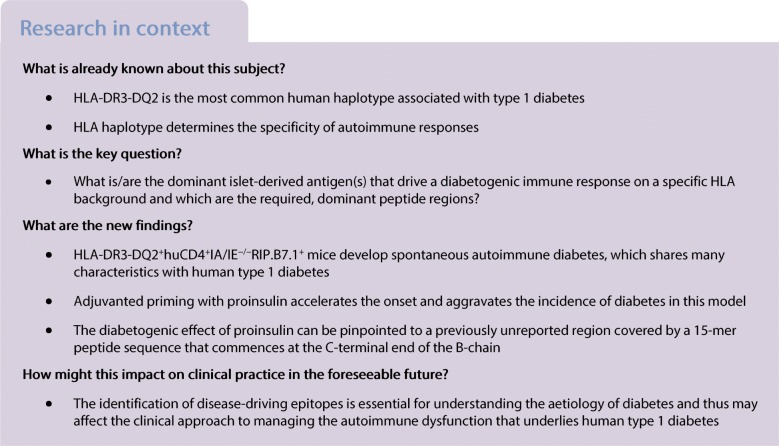



## Introduction

Type 1 diabetes, like all autoimmune diseases, results from the inappropriate activation of the immune system in response to autoantigen encounter. Although the condition can be managed increasingly well clinically, it still impacts negatively on quality of life and life expectancy [1, 2]. The reasons for the loss of self-tolerance in type 1 diabetes remain debated but it is well established that the HLA system constitutes a major genetic risk factor. HLA-DR3-DQ2 (DRB1*03:01-DQA1*05:01-DQB1*02:01) is the most common haplotype found in individuals with type 1 diabetes, at a frequency of around 34% [3], and thus delineates a major, identifiable disease cohort. Given the antigen-presenting properties of HLA molecules, it is assumed that the diabetes risk associated with HLA-DR3-DQ2 relates to the selective presentation of peptide epitopes of potentially diabetogenic autoantigens. Therefore, identification of these autoantigens and specific disease-determining regions is an important step towards understanding disease aetiology.

Attempts to discover disease-relevant epitopes in clinical studies generally involve the elution or prediction of peptides bound to candidate HLA molecules on antigen-presenting cells and/or in vitro examination of T cell responses in individuals with type 1 diabetes. This approach has generated several potentially relevant HLA-DR3-DQ2-restricted epitopes from a number of islet antigens, including GAD65 and islet antigen-2 (IA-2) [4]. Interestingly, proinsulin, generally considered to be involved in the early stages of type 1 diabetes in both humans and mice [5, 6], has not been identified as a robust source of diabetes-related epitopes presented by HLA-DR3-DQ2. One recent study, however, has highlighted regions of proinsulin C-peptide as generating HLA-DQ2-restricted CD4^+^ T cell responses in individuals with type 1 diabetes [7]. As an alternative approach to understanding autoantigen–HLA interactions, The Environmental Determinants of Diabetes in the Young (TEDDY) study has investigated the natural history of the emergence of autoantibodies on specific HLA backgrounds, revealing that in HLA-DR3-DQ2 individuals GAD65 tends to be the first of the studied islet antigens against which autoantibodies are formed, while in HLA-DR4-DQ8 individuals it is insulin [8, 9]. These studies potentially provide important clues on early events related to loss of immunological tolerance during the development of type 1 diabetes but are less incisive regarding questions of antigen and epitope diabetogenicity.

To begin to address this issue more directly, we have opted for an in vivo approach to epitope discovery, using diabetes-susceptible, HLA-transgenic mice. We examined disease relevance of antigens and peptides by their ability to promote the development of autoimmune diabetes in susceptible animals. The HLA-DR3-DQ2-transgenic mouse that we used as our base does not develop diabetes spontaneously [10], so we sought a genetic modification that would augment disease susceptibility. Moreover, through the application of adjuvanted antigen priming as a mechanism to accelerate and extend diabetes penetrance, we sought to demonstrate the importance of specific islet antigens as a diabetogenic driver on the HLA-DR3-DQ2 background.

## Methods

### Animals

HLA-DR3-DQ2-transgenic mice (C57BL/6-*H2*^*dlAb1-Ea*^Tg(Cd4-CD4)2362Litt Tg(HLA DR3-DQ2)303, described previously [10], and obtained from J. McCluskey [University of Melbourne, VIC, Australia]) were crossed with RIP-B7.1-transgenic mice (B6.Cg-Tg(*Ins2*-CD80)3B7Flv/Orl; ID 00216; EMMA, Orleans, France) in order to obtain HLA-DR3-DQ2^+^huCD4^+^IA/IE^−/−^RIP.B7.1^+^ mice, hereafter referred to as DR3DQ2×RIP-B7.1. DR4×RIP-B7.1 mice, generated by crossing B6.129S2-*H2-Ab1*^*tm1Gru*^ Tg(HLA-DRA/H2-Ea,HLA-DRB1^*^0401/H2-Eb)1Kito mice (Taconic, Germantown, MD, USA) with B6.Cg-Tg(*Ins2*-CD80)3B7Flv/Orl mice, were described previously [11]. All mice were kept under specific-pathogen-free conditions, in individually ventilated cages, at the King’s College London Biological Services Unit on 12 h light–dark cycles with food and water provided ad libitum. Experiments were conducted in accordance with UK Home Office regulations under a project licence held by M. Peakman. All work was subject to assessment and approved locally by Guy’s Animal Welfare and Ethical Review Board (AWERB).

### Priming antigens

All murine proinsulin-2 peptides were custom manufactured by either Almac (Edinburgh, UK) or GLS Biochem (Shanghai, China) at >95% purity. The 377-amino-acid C-terminal fragment of human IA-2 was produced by ProteoGenix (Schiltigheim, France). Recombinant human GAD65 (T cell GAD) was purchased from Diamyd Medical (Stockholm, Sweden). Predicted HLA binding cores and affinities were generated with the online IEDB analysis resource (http://tools.immuneepitope.org/mhcii/), using the NetMHCIIpan prediction method. Values given represent results as on 3 June 2019.

### Induction and monitoring of diabetes

All mice were monitored regularly for unprovoked glycosuria using Diastix strips (Bayer, Basel, Switzerland). Some mice were monitored weekly for hyperglycaemia, from the age of 6–7 weeks, by a minimal puncture of the tail vein at alternate sides of the tail and analysis using a OneTouch Verio meter and strips (Lifescan, High Wycombe, UK), in order to closely monitor progression towards spontaneous diabetes. Mice were considered diabetic following a blood glucose reading >16.7 mmol/l (300 mg/dl) in addition to confirmed glycosuria. For disease acceleration experiments, mice were distributed over equal or similar sized groups as indicated to achieve a comparable spread of age and sex for each group. Experiments were not randomised or blinded. Mice (aged 6–14 weeks) were primed with 100 μg of peptides or protein in TiterMax Gold adjuvant (TiterMax, Norcross, GA, USA) s.c. at the base of the tail and received a second dose s.c. distributed over the inguinal region on day 14. Mice received 200 ng pertussis toxin (Sigma, Poole, UK) in PBS intraperitoneally (i.p.) on days 0 and 1 or 2. Mice were then monitored weekly for hyperglycaemia and for glycosuria. Immediately upon detection of diabetes, mice were euthanised humanely, according to ethical approval. No animals were excluded from the data unless they had to be culled before the end of the experiment for non-diabetes-related welfare issues (e.g. overgrooming or fighting wounds), which were rare.

### Histology

Pancreases embedded in OCT compound (Cellpath, Newtown, UK) were frozen in isopentane (Sigma) cooled with liquid nitrogen. 10 μm sections were fixed in acetone before first staining with rabbit anti-mouse insulin (Ab63820 [Abcam, Cambridge, UK], 2.5 μg/ml) in PBS 0.5% BSA, detected with Vector ImmPress anti-rabbit AP (Vector Labs, Peterborough, UK), and developed with Vector ImmPress Red. Immune cells in the tissues were stained with biotinylated antibodies (all from Biolegend, San Diego, CA, USA) to murine CD4 (clone GK1.5, which also recognises human CD4, 2.5 μg/ml), CD8 (clone 53-6.7, 2.5 μg/ml), CD11b (clone M1/70, 0.25 μg/ml), CD11c (clone N418, 0.625 μg/ml), Ly6G (clone 1A8, 0.5 μg/ml) and B220 (clone RA3-6B2, 0.625 μg/ml) in PBS 0.5% BSA for 2 h. The optimal concentration for each antibody was determined by serial dilution. Staining was detected using VectaStain R.T.U. Elite ABC reagent and DAB peroxidase substrate kit from Vector Labs. Nuclei were stained with Mayer’s haematoxylin (Sigma), before mounting slides with VectaMount (Vector Labs). Images were acquired on a Zeiss Axiovert A1 microscope using the Zen (blue edition) software supplied (Zeiss, Cambridge, UK). No cropping or alteration of the images was made. See electronic supplementary material (ESM) Methods for insulitis scoring.

### Autoantibody ELISA

Proinsulin-2 peptides or recombinant human GAD65 were coated onto Maxisorp plates (Nunc, Roskilde, Denmark) in ELISA coating buffer (eBioscience/ThermoFisher, Altrincham, UK). Diluted sera (in PBS containing 5% BSA, Sigma) or controls (mouse IgG2a anti-insulin clone ICBTACLS and isotype [ThermoFisher], mouse IgG1 anti-GAD65 clone N-GAD65 and isotype [Biolegend]) were incubated for 2 h at room temperature prior to detection with biotin–anti-mouse IgG (clone Poly4053, 0.25 μg/ml in PBS 5% BSA), streptavidin–horseradish peroxidase (1:2000 in PBS 5% BSA) and high-sensitivity TMB solution (all from Biolegend) and read at 450 nm. Titrated concentrations (1–1000 ng/ml) of the relevant control antibody on each plate were used to normalise data and assign an arbitrary unit to serum antibody levels.

### Statistical analysis

All analyses were performed with GraphPad Prism 8 software (San Diego, CA, USA). For survival graphs, the Mantel–Cox logrank test was used to assess differences over the full course of the experiments. In other experiments, student’s *t* test or Dunnett’s multiple comparison test were used for comparing two groups or more, respectively. A value of *p* < 0.05 was considered statistically significant.

## Results

### Spontaneous-onset diabetes in DR3DQ2×RIP-B7.1 mice

**Fig. 1:**
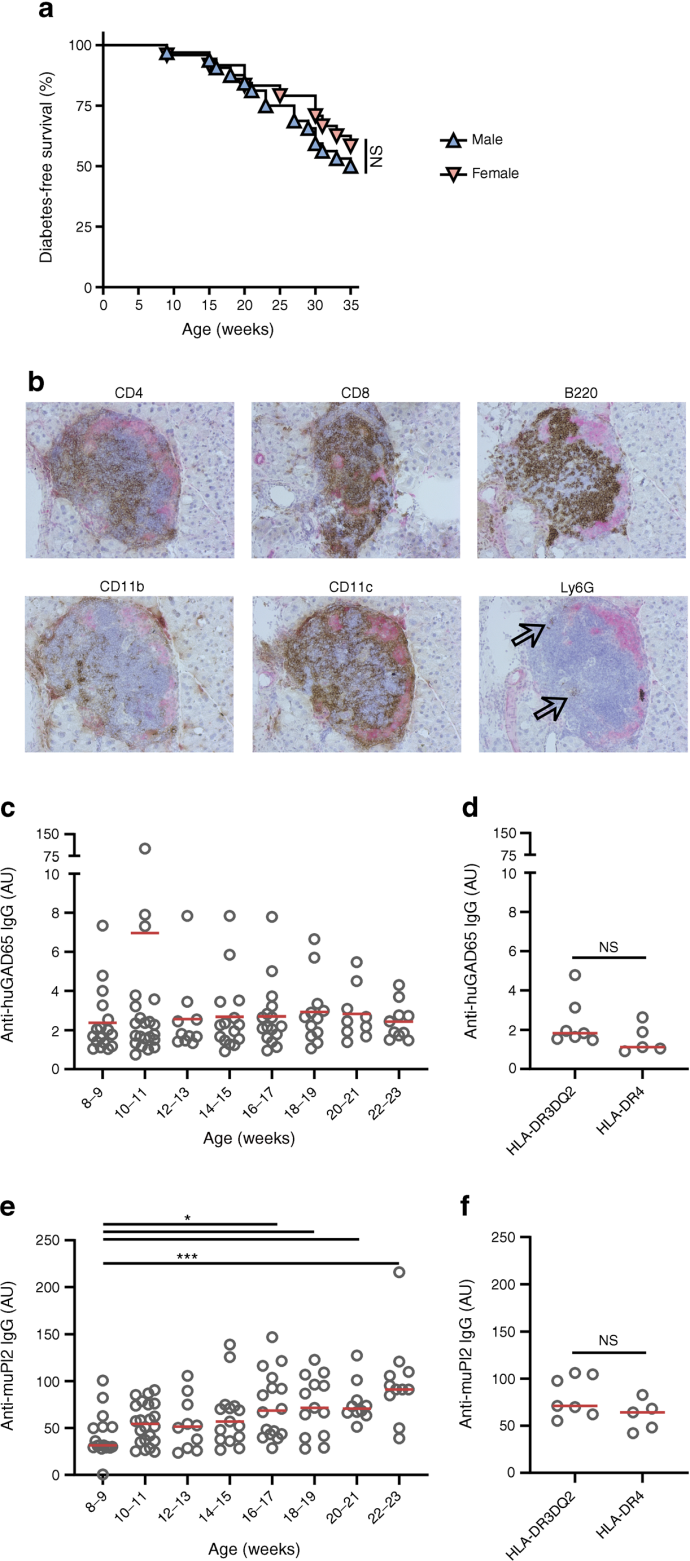
DR3DQ2×RIP-B7.1 mice develop immune-mediated diabetes spontaneously. (**a**) Mice were monitored weekly for diabetes from the age of 6–7 weeks until the age of 35 weeks (32 male, 24 female mice). Difference not significant, male vs female mice (Mantel–Cox logrank test). (**b**) Representative images of immunohistochemical double staining of frozen pancreas sections from a mouse with confirmed diabetes. Insulin stained in red, immune cells in brown. Magnification ×20. Arrows indicate Ly6G^+^ cells. (**c**–**f**) IgG autoantibody ELISAs for anti-human GAD65 (anti-huGAD65) protein (**c**, **d**) and anti-mouse proinsulin-2 (anti-muPI2) overlapping 30-mer peptides (**e**, **f**). Sera obtained cross-sectionally from mice aged 8–23 weeks were analysed (**c**, **e**). Red horizontal lines indicate median. *n* = 10–22 samples per age bracket. ELISAs were performed on the same samples, in parallel. *p < 0.05 and ***p < 0.001 (Dunnett’s multiple comparisons test). Autoantibody levels in DR3DQ2×RIP-B7.1 (HLA-DR3DQ2) mice with confirmed diabetes (*n* = 7) were compared with those in non-diabetic DR4×RIP-B7.1 (HLA-DR4) mice (*n* = 5) (**d**, **f**); not significant (Student’s *t* test). AU, arbitrary units

Unlike the DR4×RIP-B7.1 model we described previously [11], which does not show spontaneous insulitis or diabetes, DR3DQ2×RIP-B7.1 mice develop diabetes spontaneously (Fig. 1a). Although both models demonstrate relatively high baseline levels of blood glucose at any age, only DR3DQ2×RIP-B7.1 mice develop levels >16.7 mmol/l in addition to glycosuria. By the age of 35 weeks, 46% of all mice monitored (26 out of 56) had developed autoimmune diabetes. In male and female mice, disease incidence (16/32 vs 10/24, respectively) and mean ± SD age of onset were similar (24.2 ± 7.3 weeks vs 24.8 ± 8.5, respectively). No obvious signs of other immune-mediated conditions, as demonstrated by splenomegaly, cachexia, lethargy or dermal/ocular abnormalities, were detected. The transgenic human CD4, expressed in addition to the murine orthologue, did not appear to be pivotal for diabetes development (ESM Fig. 1a). All mice with diabetes demonstrated severe immune infiltrate in the pancreatic islets. This infiltrate was highly diverse, with both lymphoid and myeloid cells, expressing CD4, CD8, B220, CD11b and CD11c, found in abundance in all islets (Fig. 1b). Ly6G^+^ granulocytic cells were found in lower numbers in some islets. No substantial immune cell infiltration was observed in the non-diabetic mice that we examined histologically at the age of 6–8 weeks (*n* = 5), 10 weeks (*n* = 10), 12 weeks (*n* = 7), 16–20 weeks (*n* = 5) or even 35 weeks (*n* = 13), bar one 6-week-old male mouse in which moderate infiltration of some islets was detected (ESM Fig. 1b). This suggests that the insulitic phenotype is strongly associated with disease and perhaps reflects a rapid progression to frank diabetes. Because the onset of type 1 diabetes is characterised by the presence of autoantibodies against GAD65 and insulin in most HLA-DR3-DQ2 humans, sera of mice were tested for antibodies against murine 30-mer peptides that span the length of murine proinsulin-2, the isoform most similar to human proinsulin, and against human GAD65, which has over 95% sequence homology with the murine equivalent. As depicted in Fig. 1c, e, there was a significant increase in the levels of anti-proinsulin-2, but not anti-GAD65, antibodies when groups of mice at increasing ages were compared. There was a spread of levels of anti-GAD65 antibodies; a small number of mice displayed high binding, particularly at early ages. There did not appear to be any direct link between the appearance of these autoantibodies and the development of diabetes in individual mice. However, it should be noted that insulin autoantibodies detected in human disease typically recognise the secondary structure of insulin protein. Future work will be required to assess whether the use of conformationally intact insulin for autoantibody assays provides additional insights, such as a relationship between antibody presence/levels and disease development. Diabetic DR3DQ2×RIP-B7.1 mice displayed higher levels of autoantibodies compared with DR4×RIP-B7.1 mice that do not develop diabetes spontaneously but this difference did not reach statistical significance (Fig. 1d, f).

### Proinsulin is a diabetogenic antigen in DR3DQ2×RIP-B7.1 mice

**Fig. 2:**
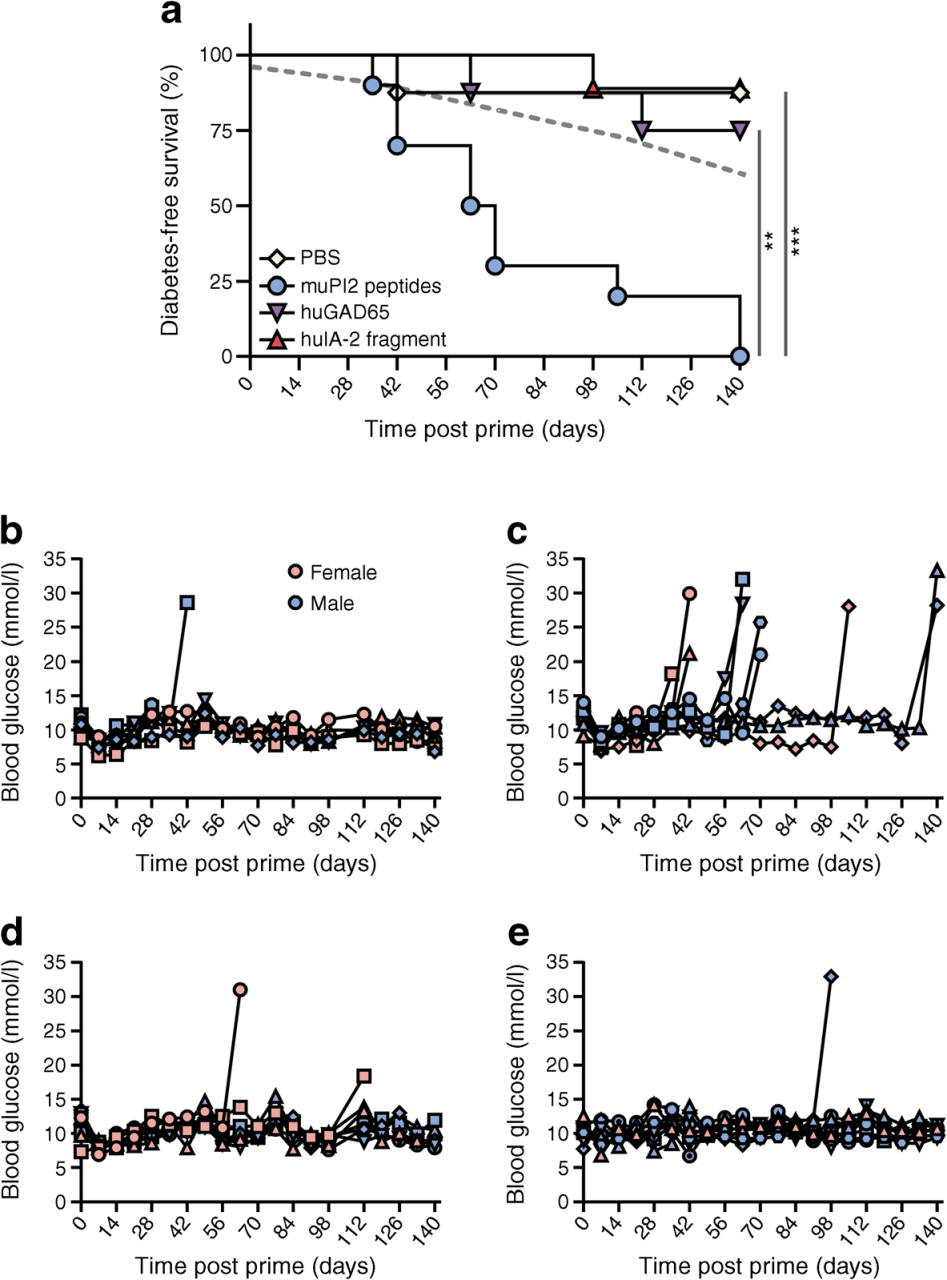
Adjuvanted priming with murine proinsulin-2 peptides promotes diabetes. (**a**) DR3DQ2×RIP-B7.1 mice aged 7–12 weeks were primed with 100 μg of murine proinsulin-2 (muPI2) peptides (6 male, 4 female mice), human GAD65 (huGAD65) protein (5 male, 3 female mice) or the 377-amino-acid C-terminal region of IA-2 (huIA-2 fragment) (7 male, 2 female mice), and a PBS only control (5 male, 3 female mice) in TiterMax Gold adjuvant s.c. on days 0 and 14, with 200 ng pertussis toxin administered i.p. on days 0 and 1 or 2. Mice were monitored weekly for diabetes up to 20 weeks post prime. The grey dashed line indicates the expected level of spontaneous diabetes in mice aged 12 weeks on day 0 of the experiments (based on the data in Fig. 1 and normalised for sex). **p < 0.01 for muPI2 peptides vs huGAD65, and ***p < 0.001 for muPI2 peptides vs both PBS and huIA-2 fragments (Mantel–Cox logrank test). (**b**–**e**) Blood glucose levels for each individual mouse in the group immunised with PBS (**b**), murine proinsulin-2 peptides (**c**), human GAD65 (**d**) or fragment of human IA-2 (**e**); key in (**b**) also applies to (**c–e**)

The spontaneous onset of diabetes in mice carrying a high-risk transgenic HLA suggests that an autoimmune process is central to disease development and prompted us to address the question of whether particular autoantigens are disease drivers. We tested the hypothesis that priming mice against candidate molecules using adjuvant would accelerate disease progression if the antigens had ‘driver’ properties. Accordingly, mice were primed with individual 30-mer peptides that overlap and span the length of murine proinsulin-2, recombinant human GAD65, the 377-amino acid intracellular region of human IA-2 or PBS alone in TiterMax Gold adjuvant (Fig. 2a–e). Importantly, of these conditions, only priming with proinsulin peptides clearly hastened diabetes onset and increased incidence beyond that observed with control stimuli or through spontaneous onset. By the end of the experiment at 20 weeks, every mouse primed with proinsulin peptides in adjuvant (10/10) had developed diabetes (mean ± SD time of onset 77 ± 38.6 days post prime). The pancreatic immune infiltrate did not differ in intensity or diversity when comparing these antigen-challenged mice with mice that developed diabetes spontaneously (not shown). These findings show the importance of immune recognition of proinsulin in the context of HLA-DR3-DQ2 as a diabetes-driving event in this model.

### A specific region of proinsulin is responsible for diabetes induction in this model

**Fig. 3:**
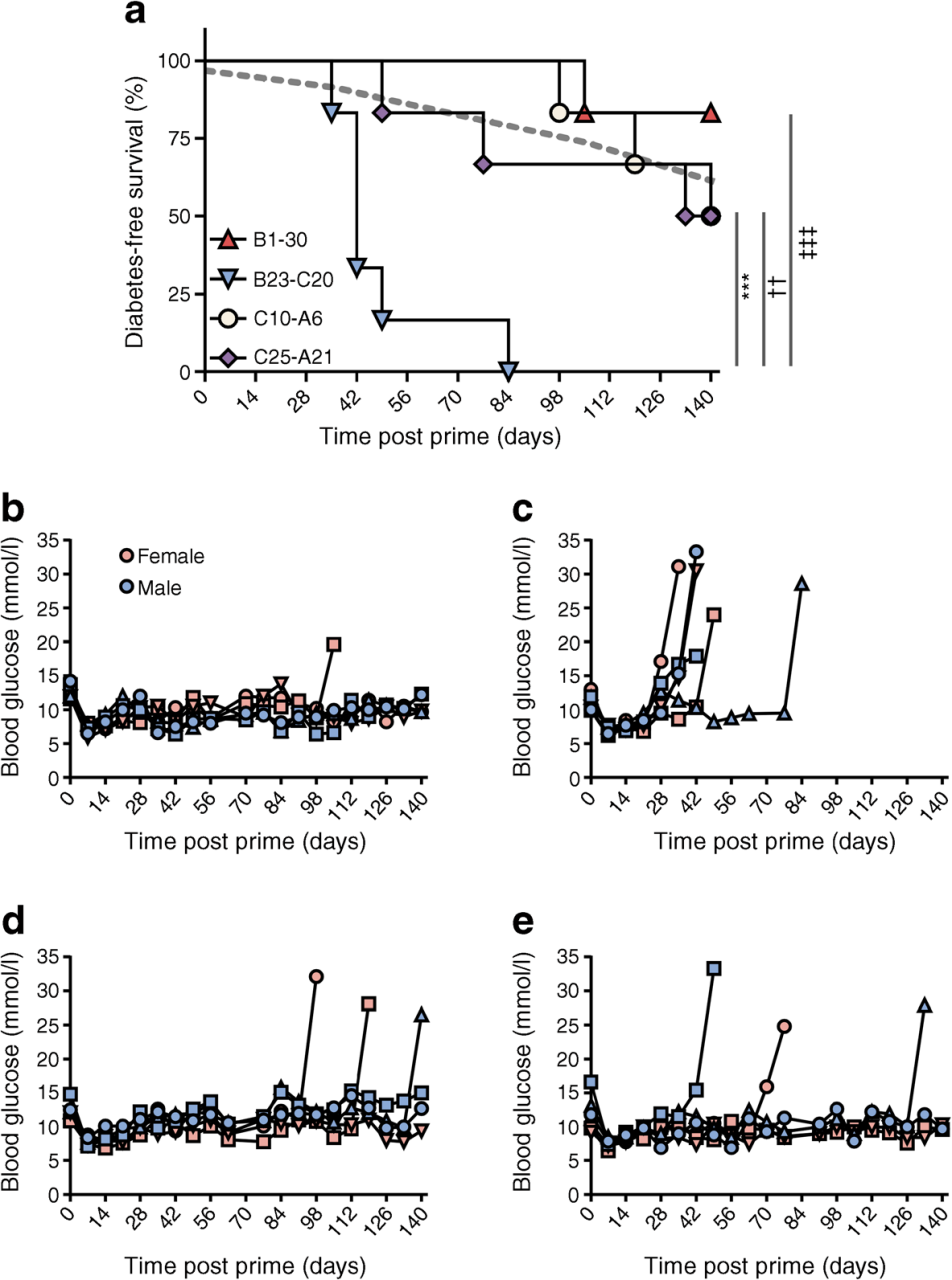
Murine proinsulin-2 peptide B23-C20 mediates diabetogenicity. (**a**) DR3DQ2×RIP-B7.1 mice aged 7–8 weeks (3 male, 3 female mice in each group) were primed with 100 μg of either murine 30-mer proinsulin-2 peptide B1-30, B23-C20, C10-A6 or C25-A21 in TiterMax Gold adjuvant s.c. on days 0 and 14, with 200 ng pertussis toxin administered i.p on days 0 and 1 or 2. Mice were monitored weekly for diabetes up to 20 weeks post prime. The grey dashed line indicates the expected level of spontaneous diabetes in mice aged 8 weeks on day 0 of the experiment (based on the data in Fig. 1 and normalised for sex). ***p < 0.001 for B23-C20 vs C10-A6; ^††^*p* < 0.01 for B23-C20 vs C25-A21; ^‡‡‡^*p* < 0.001 for B23-C20 vs B1-30; other comparisons not significant (Mantel–Cox logrank test). (**b**–**e**) Blood glucose levels for each individual mouse in the group immunised with B1-30 (**b**), B23-C20 (**c**), C10-A6 (**d**) or C25-A21 (**e**); key in (**b**) also applies to (**c–e**)

We next examined whether there is a dominant region of proinsulin that interacts in DR3DQ2×RIP-B7.1 mice to promote diabetes by repeating these adjuvanted priming experiments with each of the four proinsulin-2 peptides individually (Fig. 3a–e). Priming with B23-C20 peptide provided the most overt disease exacerbation, with very rapid development of diabetes (100% incidence by day 84 post prime; mean ± SD onset 49 ± 16.2 days post prime), characterised by severe leucocyte infiltration in the pancreatic islets (ESM Fig. 1b). In contrast, the disease incidence with the remaining three peptides was not markedly different from that expected spontaneously.

**Fig. 4:**
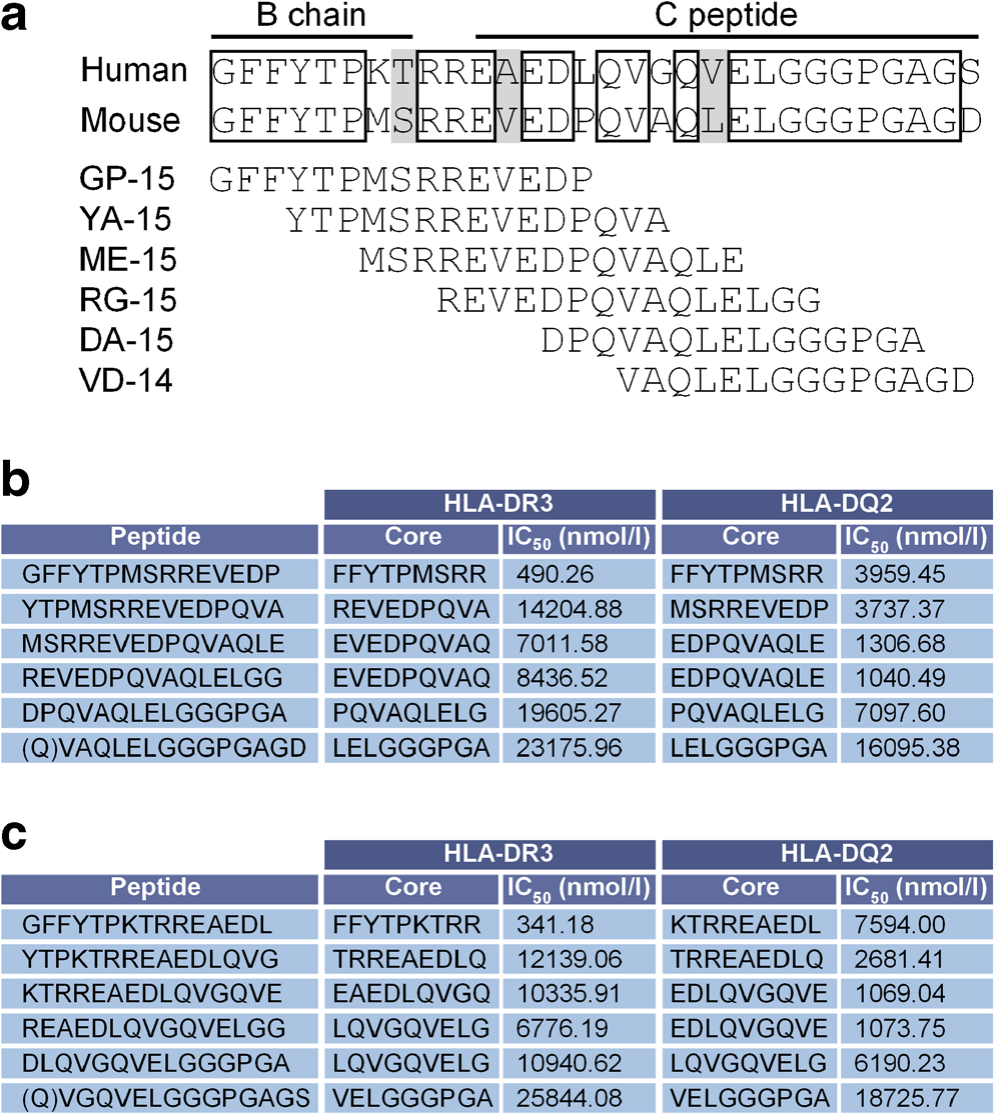
Analysis and comparison of proinsulin-2 B23-C20. (**a**) Alignment of murine proinsulin-2 with the equivalent region of human proinsulin. Boxed amino acids are identical, shaded regions are similar. Six overlapping 14- to 15-mer murine peptides generated for more detailed analysis are shown. For VD-14 peptide, the preceding glutamine (Q) in the sequence was added for the prediction, to reach the minimum 15-amino-acid length required. (**b**) Binding predictions for HLA-DR3 and HLA-DQ2 of all six murine peptides. (**c**) Binding predictions for HLA-DR3 and HLA-DQ2 of human equivalents of the murine peptides

To examine how translational these findings might be and understand potential interactions of driver antigens/epitopes with HLA, we first aligned the relevant regions of murine proinsulin-2 and human proinsulin (Fig. 4a). The B23-C20 regions of human proinsulin and murine proinsulin-2 show high homology, with 23/30 amino acids (77%) being identical and a further 3/30 (10%) similar between species. Next, to further pinpoint the region that has the dominant disease-inducing effect, we generated six overlapping 14/15-mer peptides that span the length of B23-C20. Sequences with N-terminal glutamine (Q) or glutamic acid (E) residues were avoided, to obviate formation of pyroglutamic acid, yielding peptides with unpredictable properties. One of the sequences, RG-15, was highly insoluble in aqueous solution and was therefore not incorporated in further disease-induction experiments. It has an identical predicted binding core to that of ME-15 for both HLA-DR3 and HLA-DQ2 (Fig. 4b). Based on the predicted binding affinity, none of the 14/15-mer peptides were classed as strong binders to HLA-DR3 or HLA-DQ2 by the prediction tool used. Importantly, the predicted binding core and affinity for the human equivalents of our peptides was generally comparable with mouse sequences (Fig. 4c).

**Fig. 5:**
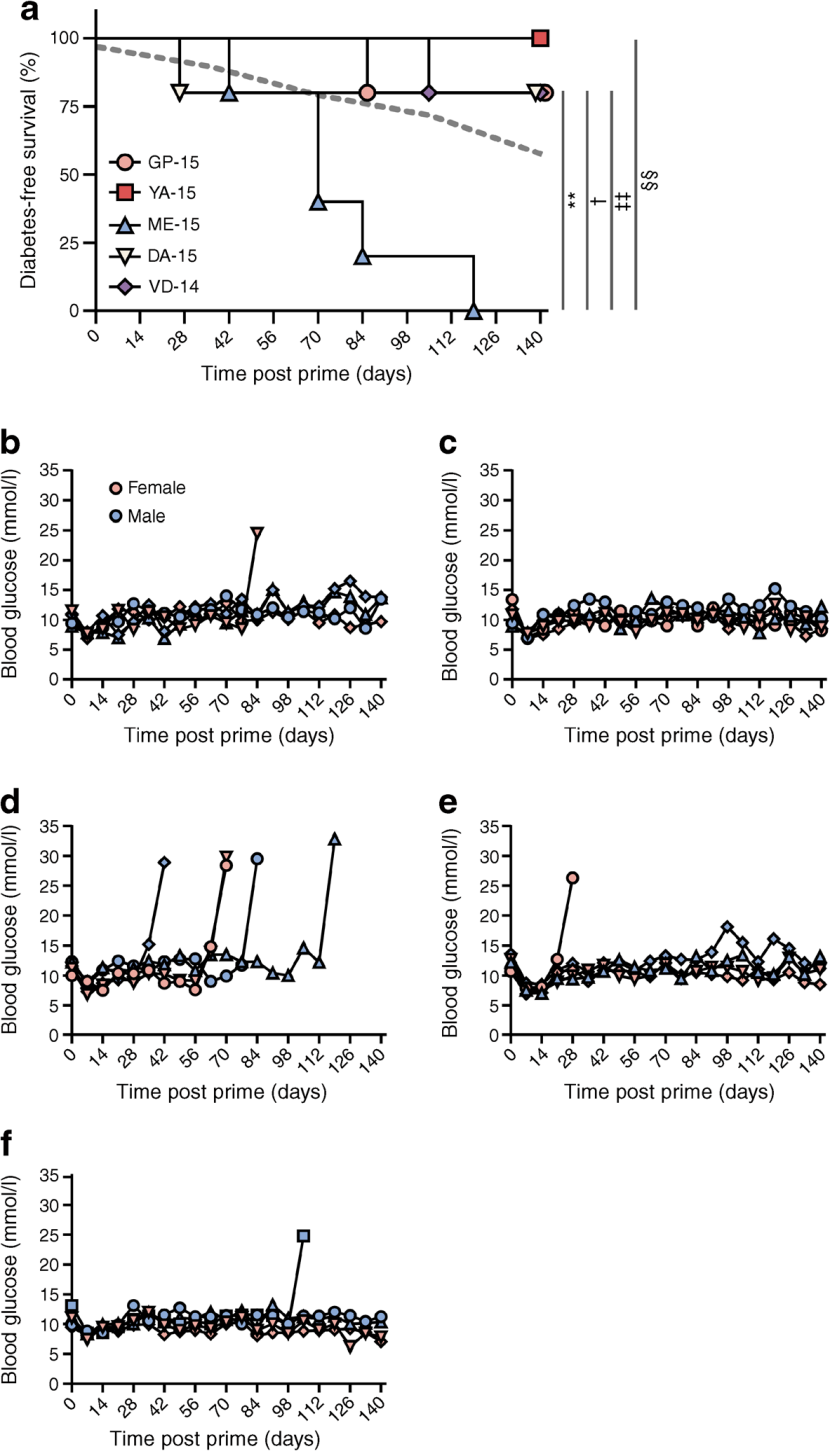
ME-15 peptide (B29-C11) covers the core region of B23-C20 for diabetogenicity. (**a**) DR3DQ2×RIP-B7.1 mice aged 10–14 weeks were primed with 100 μg of either GP-15 (2 male, 3 female mice), YA-15 (2 male, 3 female mice), ME-15 (3 male, 2 female mice), DA-15 (2 male, 3 female mice) or VD-14 (3 male, 2 female mice) peptide in TiterMax Gold adjuvant s.c. on days 0 and 14, with 200 ng pertussis toxin administered i.p on days 0 and 1 or 2. Mice were monitored weekly for diabetes up to 20 weeks post prime. The grey dashed line indicates the expected level of spontaneous diabetes in mice aged 14 weeks on day 0 of the experiments (based on the data in Fig. 1 and normalised for sex). **p < 0.01 for ME-15 vs GP-15; ^†^*p* < 0.05 for ME-15 vs DA-15; ^‡‡^*p* < 0.01 for ME-15 vs VD-14; ^§§^*p* < 0.01 for ME-15 vs YA-15; other comparisons not significant (Mantel–Cox logrank test). (**b**–**f**) Blood glucose levels for each individual mouse in the group immunised with GP-15 (**b**), YA-15 (**c**), ME-15 (**d**), DA-15 (**e**) or VD-14 (**f**); key in (**b**) also applies to (**c–f**)

Adjuvanted priming with the ME-15 (B29-C11) peptide led to markedly higher disease incidence during follow-up than would be expected spontaneously. Disease was also significantly accelerated compared with any of the other 14/15-mer peptides tested in this experiment (Fig. 5a–f), strongly suggesting that only the ME-15 peptide contains the minimum core amino acid sequence that binds HLA and interacts with immune cells to drive diabetes.

Finally, we conducted a small pilot study using an ELISPOT assay (see ESM Methods) to analyse IFN-γ secretion by human peripheral blood mononuclear cells (PBMCs) in response to the human proinsulin peptide B30-C13, which was previously reported to have the same predicted HLA-DQ binding region as B29-C11 [12]. This suggested that HLA-DR3-DQ2 donors, but not those with other HLA haplotypes, exhibit responses to B30-C13 that were greater than those to the control, HLA-DR4-restricted [13], peptide C19-A3 (ESM Fig. 2a, b). These preliminary data will require confirmation in a larger series.

## Discussion

The novel DR3DQ2×RIP-B7.1 model presented here develops diabetes spontaneously, in contrast to the DR4×RIP-B7.1 model we reported previously, which is only susceptible to induced disease [11]. Although the different approaches to the creation of these two HLA-transgenic models (chimeric vs native), and the fact that the HLA-DQ8 homologue is missing from the latter, makes direct comparison difficult, it seems likely that the inclusion of HLA-DQ2 from the DR3 high-risk haplotype is an important feature in the new model. HLA-DQ2, similar to HLA-DQ8, has previously been found to be linked strongly to diabetes susceptibility [14]. It is encouraging for the future use of inducible models of type 1 diabetes that the immune infiltrate in the pancreatic islets is very similar in the spontaneous-onset DR3DQ2×RIP-B7.1 model and the induced DR4×RIP-B7.1 model and that the diversity of immune cells infiltrating is reminiscent of human type 1 diabetes and other major preclinical models such as the non-obese diabetic (NOD) mouse [15]. The fact that we struggled to intercept pancreases at a point of moderate insulitis without established diabetes in DR3DQ2×RIP-B7.1 mice suggests that disease progression in this model may be very rapid from the point of first immune infiltration to established diabetes. Despite the fact that autoantibodies to proinsulin (and in the DR3DQ2×RIP-B7.1 model, also GAD65) could be detected, these did not appear to be directly linked to the development of diabetes in either model.

The finding that only priming with the murine proinsulin-2 peptides led to the development of diabetes was somewhat unexpected. Previous studies have predominantly identified GAD65 and IA-2 to be the target islet antigens recognised by CD4^+^ T cells or T cell clones from individuals with type 1 diabetes using the HLA-DR3 and -DQ2 restriction elements [4]. Moreover, the TEDDY study suggests that anti-GAD65 autoantibodies are typically the first to appear in disease-susceptible individuals with the HLA-DR3-DQ2 haplotype. In contrast, anti-insulin autoantibodies usually emerge first in at-risk individuals with the HLA-DR4-DQ8 haplotype [8, 9]. These represent potentially important disease endotypes, characterised by distinct pathological processes involving loss of immunological tolerance to GAD65 and insulin, respectively. In light of this, it might have been predicted that GAD65 would be the most potent antigenic driver of disease in the new DR3DQ2×RIP-B7.1 model, and yet this turned out not to be the case. There are a few caveats to our experiments, however, that should limit overinterpretation, including the fact that the GAD65 and IA-2 antigens we used (the only ones available for experimentation) are human in origin. Although highly similar to their murine orthologues, there may be critical differences within key immunogenic regions. Moreover, for IA-2 only the intracellular region, but not the full-length protein, is available as a soluble recombinant reagent. Intracellular IA-2 is known to be an important autoantigen for serological and T cell responses [13, 16], although the extracellular section also contains regions demonstrated to be immunogenic and could thus play a role in diabetes pathology [17]. Finally, protein (fragment), as used to study the response to GAD65 and IA-2, may be intrinsically less efficient than short linear peptides (e.g. as those used for proinsulin-2) at priming, breaking tolerance and driving disease. It would therefore be inappropriate to conclude categorically that GAD65 and IA-2 are not able to initiate diabetes in our model or that spontaneous disease must be mediated by targeting proinsulin alone. Nonetheless, the results are intriguing and could be consistent with there being a disconnect between the disease importance of antigens that drive early serological responses and those that drive cell-mediated beta cell destruction. Such a hypothesis could be examined in high-risk groups with particular HLA haplotypes and that are followed longitudinally from birth.

A recent study suggested that secretory and crinophagic granules of human beta cells contain only a limited array of proinsulin-derived peptides [18]. C-peptide sequences feature heavily but all of these are from the N-terminal region. N-terminal C-peptide also includes the predicted core HLA-DR3- and HLA-DQ2-binding regions of our diabetogenic ME-15 (B29-C11) peptide. It is, therefore, tantalising to speculate that the dominance in the generation of peptides from this proinsulin region by beta cells themselves may be responsible for their demise in HLA-DR3-DQ2-bearing people with type 1 diabetes. To our knowledge, peptides from the B23-C20 region of proinsulin, murine or human, have not previously been identified as being either HLA-DR3- or HLA-DQ2-restricted. Furthermore, no previous study has directly demonstrated diabetogenicity of this region in association with any haplotype; indeed, the approach of using antigen-adjuvanted priming against selected HLA backgrounds to explore the potential diabetogenicity and relative primacy of antigens and epitopes is novel as far as we are aware. For HLA-DQ8^+^ individuals, or the structurally highly similar I-A^g7^ MHC molecule in NOD mice, the B9-23 region of proinsulin is potentially important for diabetes induction [19]. In contrast, in our DR4-RIP-B7.1 model we demonstrated that disease could only be induced with proinsulin C25-A21 peptide [11], which represents a region known to be immunogenic in human type 1 diabetes [13]. Thus, where appropriate studies have been performed, a consensus on the HLA-specific immunogenic regions of proinsulin is emerging and it remains highly plausible that the B–C junction is important for HLA-DR3/DQ2-associated disease. Few studies have examined T cell reactivity to this region, and fewer still for HLA-DR3/DQ2 restricted responses. Semana et al previously found HLA-DR-restricted CD4^+^ T cell responses to proinsulin peptide C3-16, which overlaps significantly with the region we identify here, in individuals with diabetes but these were not analysed for HLA [20]. In autoantibody-positive individuals of the HLA-DRB1*04DQB1*0302 haplotype, the long B11-C24 peptide is recognised frequently [21]. So et al identified CD4^+^ T cell responses to C2-11 and C3-14 in three out of their 22 T cell clones, albeit with HLA-DQ8 restriction [7]. HLA-DQ2-restricted peptides able to generate a T cell response were all located near the C-terminal end of C-peptide. As discussed by the authors of that study, the limitation of such studies is that T cell reactivity does not necessarily imply diabetogenicity in vivo.

The new model provides opportunities to gain deeper insight into the pathology and enhance translatability. First, it will be important to identify and characterise antigen-specific CD4^+^ T cells in both spontaneous and accelerated disease. Second, additional modifications to the model could be introduced to potentially augment humanisation (e.g. by transgenic replacement of murine proinsulin with its human equivalent, allowing the direct use of human proinsulin sequences to drive the pathological processes).

In summary, we here provide in vivo evidence for the identification of a disease-relevant region within proinsulin that could have ramifications for understanding disease development in individuals with type 1 diabetes and bearing the most common HLA haplotype, HLA-DR3-DQ2.

## Electronic supplementary material

ESM (PDF 315 kb)

## Data Availability

All relevant data are available in this article and ESM. Reasonable requests for access to the in vivo models described can be made to the corresponding author.

## Abbreviations

IA-2: Islet antigen-2
RIP: Rat insulin promoter
TEDDY: The Environmental Determinants of Diabetes in the Young
