# Supplementary material for: Proinsulin peptide promotes autoimmune diabetes in a novel HLA-DR3-DQ2-transgenic murine model of spontaneous disease
Source: Diabetologia. 2019 Oct 14;62(12):2252–61. doi: 10.1007/s00125-019-04994-8 (PMC6861537; doi:10.1007/s00125-019-04994-8)
Supplement: Supplementary file 1 — (PDF 315 kb) [file 125_2019_4994_MOESM1_ESM.pdf]

Electronic Supplementary Material

ESM Methods

Insulitis scoring Frozen pancreas sections (10 μm) were stained with anti-CD45-biotin (clone A20 [Biolegend, San Diego, CA, USA], 0.25 μg/ml) in PBS 0.5%BSA), which was detected using ABC reagent and DAB solution from Vector Labs (Peterborough, UK). Nuclei were stained with Mayer's haematoxylin (Sigma, Poole, UK), before mounting slides with VectaMount (Vector Labs). At least 10 islets per mouse were assessed for leukocyte infiltration using the following ranking: 0, no infiltration; 1, low-level peri-islet presence of leukocytes around some (<50% of) islets; 2, peri-islet presence of leukocytes around >50% of islets; 3, moderate (<50% of total area) intra-islet infiltration of some islets; 4, moderate intra-islet infiltration of all islets; 5, severe (>50% of total area) infiltration of all islets.

ELISPOT assays ELISPOT assays were carried out using human peripheral blood mononuclear cells (PBMCs) isolated from fresh heparinised blood. Informed consent was acquired from all participants. $1x10^6$ PBMCs divided equally over 3 wells of a 96-well plate were stimulated for 48 hours with human proinsulin B30-C13 (TRREAEDLQVGQVELG) or C19-A3 (GSLQPLALEGSLQKRGIV) peptide (both used at 10 μg/ml and custom manufactured by ThermoFisher Scientific [Loughborough, UK]), or diluent only in RPMI media supplemented with 10% (w/v) human AB serum (Sigma). The cytokine secretion assay was performed using the IFN-γ ELISPOT kit (U-CyTech, Utrecht, The Netherlands) according to the manufacturer's instructions and plates were analysed using an automated ELISPOT Bioreader 6000 (Bio-Sys, Karben, Germany) and relevant software. Data are expressed as the total number of spots in triplicate wells after peptide stimulation divided by the total number of spots in diluent only control wells (stimulation index [SI]).

ESM Figures

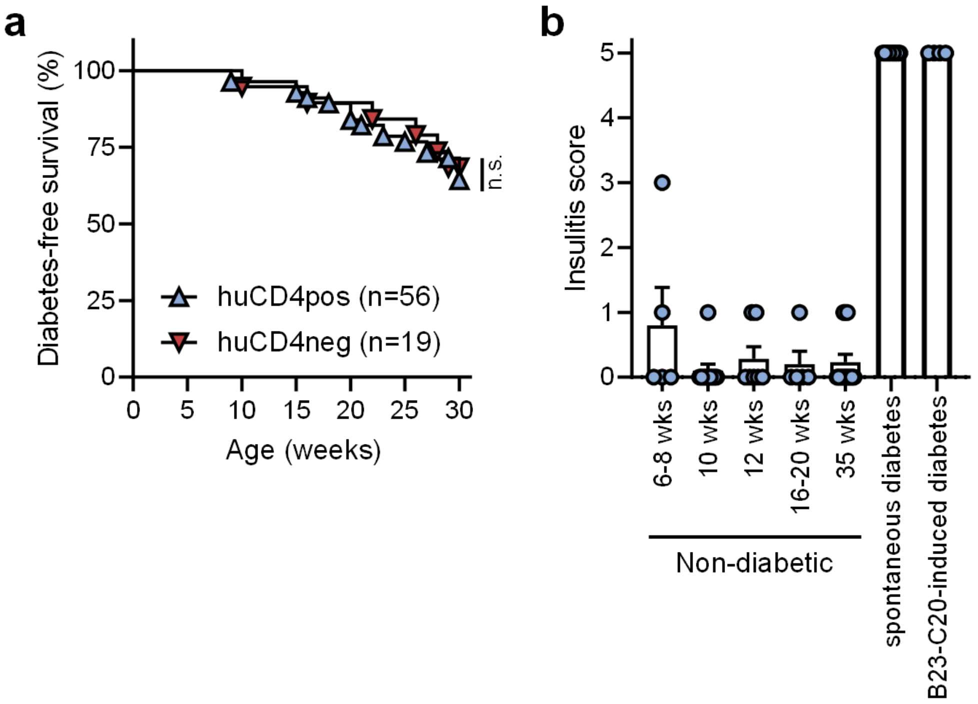

ESM Fig. 1 Further characteristics of spontaneous autoimmune diabetes in the DR3DQ8xRIP-B7.1 model. (**a**) DR3DQ2xRIP-B7.1 mice that either express (32 ♂ 24 ♀) or lack (9 ♂ 10 ♀) transgenic human CD4 were monitored for spontaneous onset diabetes from the age of 6-7 weeks until they were 30 weeks old. Difference not significant (Mantel-Cox Logrank test). (**b**) Pancreata of non-diabetic DR3DQ2xRIP-B7.1 mice aged 6-8 (n=5), 10 (n=10), 12 (n=7), 16-20 (n=5) or 35 (n=13) weeks as well as mice with spontaneous diabetes (n=9) or diabetes accelerated by priming with proinsulin B23-C20 in adjuvant (n=4), were examined for leukocytic islet infiltrate. Each dot represents one individual animal. Columns and error bars represent mean + SEM

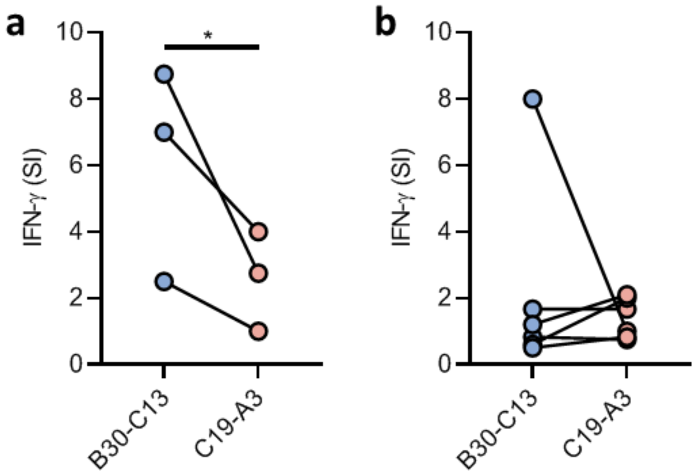

ESM Fig. 2 Responses to proinsulin B30-C13 in human donors. PBMC from individuals with either HLA-DR3-DQ2 (**a**) or any other haplotype (**b**) were stimulated *in vitro* with human proinsulin-derived peptide B30-C13 or C19-A3 prior to detection of IFN-γ by ELISPOT assay. HLA-DR3-DQ2 individuals, $n = 3$; non-HLA-DR3-DQ2, $n = 6$. * $p < 0.05$ (ratio paired t test)
